# Nontyphoidal *Salmonella* Infection Associated with Subsequent Risk of Hematological Malignancies: A Nationwide Population-Based Cohort Study

**DOI:** 10.3390/ijerph191912943

**Published:** 2022-10-10

**Authors:** Chih-Hui Yun, Wei-Chun Kao, Chung Y. Hsu, Renin Chang, Ming-Fang Cheng, Yao-Min Hung

**Affiliations:** 1 Department of Pediatrics, Kaohsiung Veterans General Hospital, Kaohsiung 813414, Taiwan; 2 Department of Medical Education and Research, Kaohsiung Veterans General Hospital, Kaohsiung 813414, Taiwan; 3 Graduate Institute of Biomedical Sciences, China Medical University, Taichung 404328, Taiwan; 4 Department of Emergency Medicine, Kaohsiung Veterans General Hospital, Kaohsiung 813414, Taiwan; 5 Department of Recreation Sports Management, Tajen University, Pingtung 907101, Taiwan; 6 Institute of Biotechnology and Chemical Engineering, I-Shou University, Kaohsiung 84001, Taiwan; 7 School of Nursing, Fooyin University, Kaohsiung 831301, Taiwan; 8 School of Medicine, National Yang Ming Chiao Tung University, Taipei 112304, Taiwan; 9 Department of Internal Medicine, Kaohsiung Municipal United Hospital, Kaohsiung 804051, Taiwan; 10 College of Health and Nursing, Meiho University, Pingtung 912009, Taiwan

**Keywords:** NTS, nontyphoidal salmonellosis, hematological malignancies, lymphoma, multiple myeloma, leukemia

## Abstract

This study aimed to investigate the relationship between nontyphoidal salmonellosis (NTS) and new-onset hematological malignancy. We conducted a 17-year nationwide, population-based, retrospective cohort study to examine the association between NTS and the risk of hematological malignancies by using the Longitudinal Health Insurance Database (LHID) of Taiwan. Participants were enrolled from 2000 to 2015 and were monitored until 2017. We traced the years 1998–2000 to ensure that the cases included were newly diagnosed with NTS. The NTS cohort included 13,790 patients with newly diagnosed NTS between 2000 and 2015. Each patient was propensity score matched at a 1:4 ratio with people without NTS. Cumulative incidence, hazard ratios (HRs), and 95% confidence intervals (CIs) were calculated after adjusting for age, sex, income, urbanization, and medical comorbidities. The adjusted hazard ratio (aHR) of hematological malignancies for NTS patients relative to those without NTS was 1.42 (95% CI 0.91–2.20). In the age subgroup analysis, NTS had a significantly greater risk of hematological malignancies for patients older than 60 (aHR 3.04, 95% CI 1.46–6.34), with an incidence rate of 11.7 per 10,000 person-years. In patients over 60 years of age, a prominent risk of hematological malignancies was observed at a follow-up of more than 3 years after the index date (aHR 3.93, 95% CI 1.60–9.65). A history of NTS is associated with the risk of subsequent hematological malignancies in Taiwanese subjects older than 60.

## 1. Introduction

Despite the advances in modern diagnostic tools and the diversity of treatment options, cancer remains the leading cause of death and results in significant lifestyle changes for patients and their families. There are many factors that may play a role in carcinogenesis, including genes, environmental carcinogens, and other medical conditions such as chronic inflammation or infection [1,2]. Infection has become an important element for cancer causation since the beginning of the 20th century. Meanwhile, more pathogens have been recognized as oncogenic, including bacteria [3,4,5]. Mechanisms that bacteria utilize include causing inflammation, producing toxins and metabolites, and reprogramming host cell signaling pathways during their life cycle [6].

Hematologic malignancy is a collective term for neoplastic diseases of the hematopoietic and lymphoid tissues and refers to three main types—leukemia, lymphoma, and myeloma. Studies have been conducted between infectious agents and hematologic malignancies, such as Epstein–Barr virus (EBV) for Burkitt’s lymphoma and human T-cell lymphotropic virus type I (HTLV-I) for adult T-cell leukemia/lymphoma (ATL) [7,8]. 

In the US, approximately 1.35 million illnesses and 420 deaths occur due to NTS annually, while 5700 infections of Salmonella Typhi occur among people in the United States each year [9,10]. In Taiwan, the average incidence of NTS-associated hospitalizations was 19.3 per 100,000 people annually [11]; however, the exact disease burden of NTS may be underestimated since most people with NTS infection may not require hospitalization.

Previous studies have revealed that nontyphoidal salmonellosis (NTS) was associated with colon, gallbladder, and gastric cancer [12,13,14,15]. However, there is a lack of discussion about NTS and other malignancies outside the gastrointestinal tract. After resisting the acidity of gastric juice and becoming a long-term inhabitant in the small and large intestines, salmonella can proliferate, penetrate the intestinal mucosa, and gradually invade the lymphoid tissues of the gastrointestinal tract [16,17]. *H*. *pylori*, which are Gram-negative bacteria that cause chronic infection in the gastrointestinal tract, have been proven to be crucial microorganisms that increase the risk of MALT lymphoma [18]. Moreover, Bascuas et al. (2018) mentioned that salmonella could be implemented as immunotherapy for non-Hodgkin lymphoma in an animal study [19]. All this evidence raised our suspicions about whether there is any relationship between NTS and hematological malignancy. To determine the potential relationship between the two topics, we conducted a 17-year nationwide retrospective cohort study to identify if people with a history of NTS would have an increased risk of hematological malignancies, including lymphoma, leukemia, and myeloma.

## 2. Materials and Methods

### 2.1. Data Source

This study was conducted using the data from the Longitudinal Health Insurance Database (LHID), a subdatabase of National Health Insurance Research Database (NHIRD). NHIRD is one of the largest medical databases in the world, which includes the claims data from Taiwan’s National Health Insurance (NHI) program.

National Health Insurance (NHI), established in 1995, is a nation-based insurance program in Taiwan. In June 2021, 23,876,603 people were participating in NHI, including over 99% of the total population in Taiwan [20].

The NHI program includes over 90% of the medical departments in Taiwan, and all the information of medical visits to these medical departments is recorded and reported to the NHI center. NHI recorded diseases based on the International Classification of Diseases, Ninth Revision, Clinical Modification (ICD-9-CM) before 2015, and ICD-10 was employed instead after 2016.

NHIRD contains the medical information of every insured person, including the patient’s basic profile, diagnosis, prescription details, laboratory examination, and procedures. To maintain data confidentiality, identifiable private information is encrypted before the data are released to researchers; however, the medical information remains unique for each insured person, providing adequate internal linkage of records in the database.

We obtained the data from 1998 to 2017 at the Health and Welfare Data Science Center, Ministry of Health and Welfare, Taiwan. This study was approved by the Institutional Review Board of China Medical University Hospital Research Ethics Committee CMUH109-REC2-031(CR-2). All the data from NHIRD were delinked from private information before release; therefore, the need for informed consent forms was waived. This study also adhered to the Declaration of Helsinki and the relevant laws in Taiwan.

### 2.2. Study Design and Population

This was a retrospective cohort study, and we identified all patients with NTS between 2000 and 2015. NTS was defined according to ICD-9-CM code 003 and ICD-10, and only patients with at least 3 relevant outpatient department visits or with history of hospital admission were enrolled in our study. The index date was defined as the date of the first diagnosis of NTS, and patients with a history of any malignancy (ICD9: 140–208) before the index date were excluded.

The non-NTS group was matched with the NTS group at a 4:1 ratio to increase precision. We implemented propensity score matching to reduce the potential bias caused by heterogenicity of the baseline characteristics of the two cohorts. Both cohorts shared the same index date and follow-up duration, and we controlled covariates including age, sex, and history of medical comorbidities in both cohorts. The medical comorbidities in the logistic regression models included diabetes (ICD-9-CM code 250, ICD-10 E08.00, E08.01, E09.00, E09.01, E11.00, E11.01, E11.65, E11.69, E11.9, E13.00, E13.01, E13.9), hypertension (ICD-9-CM codes 401–405, ICD-10 I10, I11.0, I11.9, I12.0, I12.9; I13.0, I13.1, I13.11, I13.2; I15.0, I15.1, I15.2, I15.8, I15.9, N26.2), hyperlipidemia (ICD-9-CM code 272, ICD-10 E78.0–78.6), human immunodeficiency virus (HIV) infection (ICD-9-CM codes 042–044, 795.8, and V08, ICD-10 B20), hepatitis B (ICD-9-CM codes 070.2, 070.3, V02.61), hepatitis C (ICD-9-CM codes 070.41, 070.44, 070.51, 070.54, V02.62 ICD-10 B27.90), and autoimmune disease (AID) such as systemic lupus erythematosus (SLE) (ICD-9-CM code 710.0, ICD-10 M32.10, M32.19, M32.8, M32.9), rheumatoid arthritis (ICD-9-CM code 714.0, ICD-10 M05.7), and Sjögren’s syndrome (ICD-9-CM code 710.2, ICD-10 M35). 

Both cohorts were monitored from the index year to 31 December 2017, and conditions to stop monitoring included loss of follow-up, patient death, withdrawal from insurance, or diagnosis of hematological malignancies, including any kind of lymphoma, multiple myeloma, or leukemia.

### 2.3. Outcomes

The main outcome of our study was the occurrence of hematological malignancies, which include lymphoma (ICD-9-CM codes 202.x, ICD-10 C81-C88), multiple myeloma (ICD-9-CM codes 203.x, ICD-10 C90), and leukemia (ICD-9-CM codes 204.x, ICD-10 C91–95).

In Taiwan, all patients with cancers, including hematological malignancy, are indicated to apply for Catastrophic Illness Certificates (CICs), which can provide medical expense discounts or medical charge waivers. To apply for CICs, all diagnoses of cancers require adequate histological evidence and associated laboratory or imaging findings, and all application files require mandatory peer review by NHI. The strict review by NHI experts provides sufficient reliability for the diagnosis of hematological malignancy in our study.

### 2.4. Statistical Analysis

We analyzed the data of demographic characteristics (distributions of categorical age, sex, and comorbidities) of the NTS and non-NTS groups using the Chi-square (χ^2^) test or Student’s *t*-test as appropriate. We calculated the incidence rate by the number of occurrences and person-years. We utilized hazard ratios (HRs) and 95% confidence intervals (CIs) of the two groups for estimates in univariate and multivariate Cox proportional hazard regression models. The Kaplan–Meier curve was plotted to describe the cumulative incidence between the two groups; then, the log rank test was used. SAS statistical software package version 9.1 (SAS Institute, Cary, NC, USA), was employed for our statistical analysis, and the significance level was set at 0.05.

## 3. Results

This study included 13,790 patients with NTS and 55,160 matched patients without NTS. The baseline characteristics of both groups are listed in Table 1. There were no significant differences in age, sex, and comorbidities; however, there were significant differences in several variables, including urbanization (*p* < 0.0001), follow-up time (*p* = 0.0062), the usage of proton pump inhibitor (*p* < 0.0001), sucralfate (*p* < 0.001), and NSAIDs (*p* < 0.001). We calculated SMD to interpret how these differences would affect our results, and all of them had a small effect.

**Figure 1:**
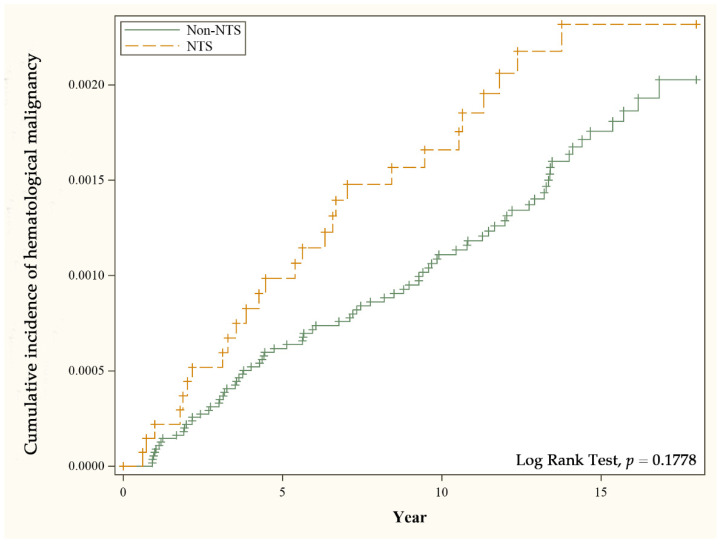
Cumulative incidence of hematological malignancy for patients with and without nontyphoidal salmonellosis.

Table 2 presents the Cox regression analyses of risk factors associated with hematological malignancy. There was no significant difference in the risk of hematological malignancy between the NTS group and the matched group. The adjusted hazard ratio (aHR) of hematological malignancy in NTS patients compared with the matched group is 1.42 (95% CI 0.91–2.20) after adjusting for age, insurance premium level, urbanization, and comorbidities. The cumulative incidence of hematological malignancy in the different cohorts is displayed in Figure 1.

**Figure 2:**
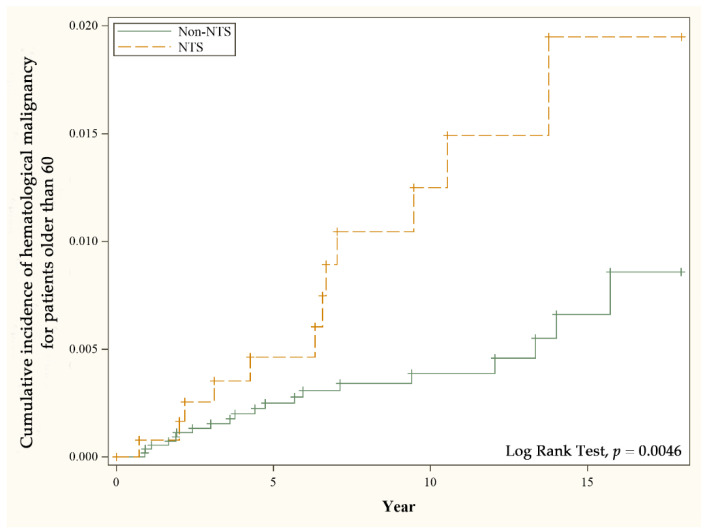
Cumulative incidence of hematological malignancy for patients older than 60 with and without nontyphoidal salmonellosis.

A comparison of the incidence of hematological malignancy between NTS and non-NTS groups in different characteristics is shown in Table 3. The age subgroup analysis revealed a significantly greater risk of hematological malignancy in patients older than 60 with NTS (aHR 3.04, 95% CI 1.46–6.34, *p* value 0.0030) compared with the same age subgroup. The incidence rate is 11.7 per 10000 person-year, and the cumulative incidence of hematological malignancy in different cohorts in this age subgroup is displayed in Figure 2.

In patients older than 60, the elevated risk of hematological malignancy was more prominent when the follow-up time was >3 years after the NTS episode (aHR 3.93, 95% CI 1.60–9.65), compared with non-NTS patients in the same age subgroup and follow-up period (Table 4). In the subgroup analysis of patients with different comorbidities, patients with NTS and hypertension had an elevated risk of hematological malignancy compared with the matched group without NTS (aHR 4.06, 95% CI 1.99–8.31).

## 4. Discussion

To our knowledge, this 17-year population-based retrospective cohort study is the first to investigate the epidemiologic association between nontyphoidal salmonella infection and the subsequent risk of hematological malignancies. We identified nontyphoidal salmonella infection as an independent risk factor for developing hematological malignancies among patients older than 60 (adjusted HR 3.04, 95% C.I. 1.46 to 6.34) after adjusting for the baseline demographic characteristics and comorbidities.

Most of the pathogeneses of hematological malignancy were found to be related to genetic abnormalities, such as chromosomal translocation or genetic mutation. These chromosomal or genetic abnormalities may be related to several conditions that can cause DNA damage, including radiation exposure, chemical exposure, or chronic inflammation with infective or non-infective causes [21].

Increasing evidence has revealed that many different infectious agents play certain roles in specific cancers, such as EBV in Burkitt’s lymphoma and Hodgkin’s disease, *H*. *pylori* in gastric cancer, or NTS in gallbladder cancer, and these pathogens vary with the possible mechanisms of oncogenesis [22,23].

NTS results in acute, self-limited disease most of the time; however, NTS has been known to colonize the human gastrointestinal tract, and continued shedding of bacteria for a short or prolonged period has been documented. The carriage period in a human host can last for weeks to months, but longer carriage periods for years have also been reported [24].

NTS can continue to grow in the human body when the host’s immune system fails to clear the bacteria entirely, resulting in chronic carriage without clinical symptoms [25]. NTS can survive in human hosts with several immune escape mechanisms, such as immune modulation or biofilm formation [26,27]. The clinical symptoms may be absent during salmonella carriage, but the effects of immuno-modulation and chronic carriage may result in chronic inflammation, which has been demonstrated to be closely associated with most types of cancer [28,29].

In our study, there was no significant difference in the risk of hematological malignancy between the NTS and matched cohorts, but the age subgroup analysis revealed a significantly elevated risk of hematological malignancy in patients older than 60 with NTS (aHR 3.04, 95% CI 1.46–6.34, *p* value 0.0030) compared with the matched age subgroup. Previous evidence demonstrated that older individuals might have a higher risk for prolonged salmonella carriage [30], which may place the host at greater risk of chronic inflammation. The higher risk of chronic inflammation in older patients may be a possible reason explaining why the elevated risk of hematological malignancy was only found in patients older than 60 with NTS, although the relationship between chronic inflammation and hematological malignancy still needs to be determined.

In the group of patients older than 60 with NTS, we found that patients with a longer follow-up time (>3 years) after an NTS episode had an even greater risk of hematological malignancy (aHR 3.93, 95% CI 1.60–9.65) compared with the matched subgroup. There are two possible explanations for these results. First, a longer salmonella carriage period may correlate with a longer period of chronic inflammation, which may be one of the risk factors for oncogenesis. Second, it takes time for individuals to progress from the oncogenic process to develop detectable clinical symptoms for malignancy. 

In the subgroup analysis in patients with different comorbidities, we found that patients with hypertension and NTS had an elevated risk for hematological malignancy (aHR 4.06, 95% CI 1.99–8.31) compared with the matched group without NTS. Hypertension was found to be a risk factor in many types of cancer, including kidney, colorectal, breast, endometrial, liver, and esophageal cancer [31]. According to our literature review, there is no significant evidence regarding the relationship between hypertension and the development of hematological malignancy, and more studies are needed in the future to evaluate this relationship.

### Strength and Limitation

Our study was conducted using the LHID, a subdatabase of NHIRD, which collects data from the NHI program, a nationwide initiative covering over 99% of the total population in Taiwan. The implications of confounding factors were controlled by propensity score matching with similar cohorts. 

Our study also has several limitations. First, despite the large number of patients enrolled in our study, there were few cases that developed hematological malignancy. This may be due to the low prevalence of hematological malignancy; however, the implications of the low number of cases on our analytical results cannot be ignored. Second, we evaluated NTS and the potential risk of hematological malignancy; however, the relationship and possible mechanisms between them still needs to be determined. Third, it is not clear whether the hematologic malignancy developed in the study group was specifically due to NTS. Although there might have been genetic changes, mutations, or triggers from the microenvironment that were responsible for such a transformation, there is a lack of direct evidence.

## 5. Conclusions

People older than 60 with NTS may have a greater risk of hematological malignancy, and patients older than 60 who have an NTS episode and follow-up time of more than 3 years have an even greater risk of hematological malignancy compared with the matched age group. Considering the large sample size and relatively modest effects, these findings should be replicated in other datasets or countries. 

## Figures and Tables

**Table 1:** Characteristics of patients with and without NTS in Taiwan.

Variables	Non-NTS, *n* (%)	NTS, *n* (%)	*p*-Value	SMD
All	55,160 (100)	13,790 (100)		
Sex			0.7519	0.003
Female	27,583 (50.01)	6875 (49.85)		
Male	27,577 (49.99)	6915 (50.15)		
Age (year)			0.9593	
<40	41,970 (76.09)	10,487 (76.05)		0.001
≥60	5696 (10.33)	1435 (10.41)		0.001
40–59	7494 (13.59)	1868 (13.55)		0.003
Mean ± SD	22.40 ± 23.58	22.12 ± 23.93	0.2097	0.012
Insurance premium level (NTD)			0.9754	
<20,000	33,772 (61.23)	8455 (61.31)		0.002
20,000–39,999	15,192 (27.54)	3785 (27.45)		0.002
≥40,000	6196 (11.23)	1550 (11.24)		<0.0001
Urbanization			<0.0001	
1 (most urbanized)	30,979 (56.16)	7428 (53.87)		0.046
2	19,161 (34.74)	4584 (33.24)		0.032
3	5020 (9.10)	1778 (12.89)		0.121
Comorbidities				
Hypertension	6102 (11.06)	1526 (11.07)	0.9903	<0.0001
Diabetes	3376 (6.12)	856 (6.21)	0.7034	0.004
Hyperlipidemia	4315 (7.82)	1070 (7.76)	0.8038	0.002
Autoimmune disease	1190 (2.16)	303 (2.20)	0.7735	0.003
HCV	424 (0.77)	113 (0.82)	0.5442	0.006
HIV	148 (0.27)	45 (0.33)	0.2488	0.011
Infectious mononucleosis	63 (0.11)	20 (0.15)	0.3505	0.009
Medication				
PPI	439 (0.80)	260 (1.89)	<0.0001	0.095
Sucralfate	30 (0.05)	24 (0.17)	<0.0001	0.035
NSAIDs	8070 (14.63)	3153 (22.86)	<0.0001	0.212
Aspirin	1564 (2.84)	405 (2.94)	0.5220	0.006
Statin	1195 (2.17)	309 (2.24)	0.5930	0.005
Metformin	1383 (2.51)	325 (2.36)	0.3092	0.010
Follow-up time	12.85 ± 4.27	12.74 ± 4.52	0.0062	0.026

Abbreviations: NTS, nontyphoidal salmonellosis; NTD, new Taiwan dollar; HCV, hepatitis C virus; HIV, human immunodeficiency virus; PPI, proton pump inhibitor.

**Table 2:** Cox regression analyses of risk factors associated with hematological malignancy among patients in Taiwan.

Variables	Event	Crude		Adjusted	
		HR (95% CI)	*p*	HR (95% CI)	*p*
NTS					
No	81	1 (Reference)		1 (Reference)	
Yes	27	1.35 (0.87, 2.08)	0.1791	1.42 (0.91, 2.20)	0.1189
Sex					
Female	48	1 (Reference)		1 (Reference)	
Male	60	1.26 (0.86, 1.84)	0.2388	1.51 (1.03, 2.21)	0.0367
Age (year)					
<40	42	1 (Reference)		1 (Reference)	
40–59	34	5.51 (3.50, 8.67)	<0.0001	5.20 (3.21, 8.42)	<0.0001
≥60	32	10.13 (6.35, 16.16)	<0.0001	8.44 (4.64, 15.35)	<0.0001
Insurance premium level (NTD)					
<20,000	72	1 (Reference)		1 (Reference)	
20,000–39,999	26	0.78 (0.50, 1.22)	0.2764	0.92 (0.58, 1.45)	0.7165
≥40,000	10	0.75 (0.39, 1.46)	0.3983	0.72 (0.37, 1.40)	0.3279
Urbanization					
1 (most urbanized)	67	1 (Reference)		1 (Reference)	
2	34	0.83 (0.55, 1.25)	0.3738	0.81 (0.53, 1.22)	0.3096
3	7	0.59 (0.27, 1.29)	0.1869	0.54 (0.25, 1.18)	0.1204
Comorbidities					
Hypertension	32	5.71 (3.75, 8.70)	<0.0001	1.54 (0.88, 2.72)	0.1340
Diabetes	13	3.54 (1.97, 6.36)	<0.0001	0.53 (0.21, 1.31)	0.1674
Hyperlipidemia	16	3.28 (1.92, 5.60)	<0.0001	0.75 (0.38, 1.49)	0.4074
Autoimmune disease	6	4.20 (1.84, 9.61)	0.0007	1.74 (0.74, 4.12)	0.2061
HCV	4	8.79 (3.22, 23.94)	<0.0001	3.03 (1.09, 8.42)	0.0340
HIV	0	NA	NA	NA	NA
Infectious mononucleosis	0	NA	NA	NA	NA
Medication					
PPI	3	5.35 (1.69, 16.90)	0.0043	1.46 (0.45, 4.74)	0.5300
Sucralfate	0	NA	NA	NA	NA
NSAIDs	23	1.54 (0.97, 2.44)	0.0677	0.98 (0.60, 1.59)	0.9191
Aspirin	6	3.30 (1.45, 7.55)	0.0046	0.66 (0.27, 1.62)	0.3681
Statin	7	5.79 (2.67, 12.54)	<0.0001	1.77 (0.68, 4.58)	0.2425
Metformin	8	5.48 (2.65, 11.32)	<0.0001	2.19 (0.75, 6.41)	0.1511

Abbreviations: HR, hazard ratio; CI, confidence interval; NTS, nontyphoidal salmonellosis; NTD, new Taiwan dollar; HCV, hepatitis C virus; HIV, human immunodeficiency virus; PPI, proton pump inhibitor.

**Table 3:** Incidence rate and hazard ratio of hematological malignancy in different stratifications.

Subgroup	Non-NTS			NTS			NTS Cohort vs. Non-NTS Cohort			
							Crude		Adjusted	
	*n*	PY	IR	*n*	PY	IR	HR (95% CI)	*p*	HR (95% CI)	*p*
Overall	81	708,842	1.14	27	175,657	1.54	1.35 (0.87, 2.08)	0.1791	1.42 (0.91, 2.20)	0.1189
Sex										
Female	37	355,235	1.04	11	88,121	1.25	1.20 (0.61, 2.35)	0.5937	1.21 (0.61, 2.39)	0.5780
Male	44	353,608	1.24	16	87,536	1.83	1.47 (0.83, 2.61)	0.1854	1.66 (0.93, 2.97)	0.0853
Age (year)										
<40	36	576,820	0.62	6	144,337	0.42	0.67 (0.28, 1.58)	0.3595	0.69 (0.29, 1.64)	0.3964
40–59	25	86,076	2.90	9	21,065	4.27	1.47 (0.69, 3.14)	0.3233	1.36 (0.63, 2.95)	0.4334
≥60	20	45,947	4.35	12	10,255	11.70	2.70 (1.32, 5.52)	0.0066	3.04 (1.46, 6.34)	0.0030
Insurance premium level (NTD)										
<20,000	56	430,604	1.30	16	105,900	1.51	1.16 (0.67, 2.03)	0.5925	1.18 (0.67, 2.06)	0.5636
20,000–39,999	19	199,216	0.95	7	49,545	1.41	1.49 (0.62, 3.53)	0.3702	1.84 (0.75, 4.50)	0.1848
≥40,000	6	79,022	0.76	4	20,213	1.98	2.58 (0.73, 9.15)	0.1420	3.38 (0.89, 12.80)	0.0725
Comorbidities										
Hypertension	18	50,484	3.57	14	11,433	12.24	3.45 (1.72, 6.94)	0.0005	4.06 (1.99, 8.31)	0.0001
Hyperlipidemia	13	36,090	3.60	3	8352	3.59	1.00 (0.28, 3.50)	0.9959	1.27 (0.36, 4.54)	0.7126
HIV	0	872	0.00	0	219	0.00	NA	NA	NA	NA
Infectious mononucleosis	0	647	0.00	0	199	0.00	NA	NA	NA	NA
Medication										
Sucralfate	0	285	0.00	0	197	0.00	NA	NA	NA	NA
NSAIDs	15	95,843	1.57	8	36,103	2.22	1.41 (0.60, 3.33)	0.4295	1.44 (0.61, 3.43)	0.4073
Aspirin	3	12,641	2.37	3	2680	11.19	4.84 (0.98, 23.98)	0.0537	4.31 (0.78, 23.72)	0.0932
Metformin	5	10,450	4.78	3	2159	13.90	3.00 (0.72, 12.55)	0.1329	3.61 (0.76, 17.21)	0.1077

Abbreviations: IR, incidence rate; HR, hazard ratio; CI, confidence interval; PY, person-years; NTS, nontyphoidal salmonellosis; NTD, new Taiwan dollar; HIV, human immunodeficiency virus.

**Table 4:** Incidence rate and hazard ratio of hematological malignancy in patients older than 60 with different follow-up times in Taiwan in 2000–2017.

Subgroup	Non-NTS			NTS			NTS Cohort vs. Non-NTS Cohort			
							Crude		Adjusted	
	*n*	PY	IR	*n*	PY	IR	HR (95% CI)	*p*	HR (95% CI)	*p*
Follow-up time (year)										
<3	7	15,808	4.43	3	3605	8.32	1.90 (0.49, 7.33)	0.3537	1.92 (0.49, 7.57)	0.3520
≥3	13	30,139	4.31	9	6650	13.53	3.14 (1.34, 7.34)	0.0084	3.93 (1.60, 9.65)	0.0029

Abbreviations: IR, incidence rate; HR, hazard ratio; CI, confidence interval; PY, person-years; NTS, nontyphoidal salmonellosis; NTD, new Taiwan dollar; HIV, human immunodeficiency virus.

## Data Availability

The data presented in this study are contained within the article.
